# Recovery of Platinum Group Metals from Spent Automotive Catalysts: A Review of Processes and Challenges

**DOI:** 10.3390/ma19122491

**Published:** 2026-06-10

**Authors:** Minghui Liu, Chunzhen Yang, Ming Tian, Yutong Zhao, Xianghui Liu, Chenyu Zhan, Zihan Li, Tianyan Xue, Faquan He, Hongliang Wang, Jianhui Yang

**Affiliations:** 1 China Energy Longyuan Environmental Protection Co., Ltd., Beijing 100039, China; 2 National Engineering Research Center of Green Recycling for Strategic Metal Resources, Institute of Process Engineering, Chinese Academy of Sciences, Beijing 100190, China; 3 Efficient Electrolysis Group, Fuel Cell System and Engineering Laboratory, Dalian Institute of Chemical Physics, Chinese Academy of Sciences, Dalian 116023, China

**Keywords:** platinum group metals, resource recycling, spent automotive catalysts, Fe−Si−PGMs alloy, PGM analytical methods

## Abstract

Platinum group metals (PGMs: Pt, Pd, Rh, Ru, Os, Ir) are critical strategic metals. Spent automotive catalysts (SACs) represent one of the most significant secondary sources of PGMs, and their recovery is essential for alleviating the supply–demand imbalance. In the recycling chain, pyrometallurgical processing of SACs generates Fe-Si-based alloy concentrates (termed Fe−Si−PGMs), serving as an important yet challenging intermediate resource for PGM recovery. This review first summarizes the pyrometallurgical and hydrometallurgical processes used for recovering PGMs from SACs, before shifting its focus to the treatment technologies for PGMs in Fe–Si–PGMs alloy. These techniques, including direct extraction, extraction following desilication (via alkaline roasting, slagging, or hydrometallurgical routes), and in situ mechanochemical extraction, are critically evaluated in terms of their advantages and limitations. Furthermore, given that the accurate quantification of trace-level yet high-value PGMs represents another key challenge in the recovery chain due to complex sample matrices, this work systematically outlines and compares the analytical methods commonly employed, such as fire assay, spectroscopic and mass spectrometric techniques, electrochemical methods, and alkali fusion. Finally, several recommendations are provided regarding PGM recovery from SACs, with emphasis on Fe−Si−PGMs alloy processing and analytical methods for PGMs.

## 1. Introduction

Accelerating global economic growth and industrialization have intensified the dependence of human society on natural resources, particularly critical metals. Among these, platinum group metals (PGMs: Pt, Pd, Rh, Ir, Os, Ru) are indispensable owing to their unique physicochemical properties, such as exceptional catalytic activity, high-temperature stability, superior corrosion resistance, and excellent electrical conductivity. PGMs serve not only as the core materials for automotive catalysts but are also extensively utilized in critical sectors, including petrochemicals, electronics, fuel cells, biomedicine, aerospace, and high-end jewelry [1,2]. However, as one of the rarest categories of metallic elements on Earth, PGMs possess extremely limited global reserves and a highly concentrated geographical distribution. Global PGM reserves are estimated at approximately 70,000 tonnes, primarily located in only a few countries, such as South Africa, Russia, and Zimbabwe. South Africa alone accounts for about 90% of the global total [3,4]. This pattern of highly concentrated resource distribution, coupled with the intrinsic scarcity of PGMs, the environmental pressures associated with mining, and geopolitical uncertainties, subjects the primary supply to persistent and severe challenges. This results in volatile market prices and poses significant risks to the stability of related global supply chains [5]. Consequently, the recycling and recovery of PGMs from secondary resources are of paramount importance.

Among all application fields of PGMs, the automotive industry undoubtedly represents the largest consumer sector. Spent automotive catalysts (SACs), whose active components are Pd, Pt, and Rh, constitute one of the most significant secondary resources for PGMs. The PGM content in SACs can be hundreds of times higher than that in primary ores, earning them the designation “mobile PGM mines” [3,6]. Furthermore, SACs contain various soluble hazardous substances. If disposed of simply through stockpiling or landfilling, they pose a potential risk of causing severe environmental contamination. Therefore, recovering PGMs from SACs not only effectively alleviates the pressure on mining primary mineral resources, enhances supply chain stability, and reduces material acquisition costs but also carries significant positive implications for environmental protection and sustainable development [7]. The processes for recovering PGMs from SACs can be broadly categorized into two major types: pyrometallurgy and hydrometallurgy. Each category possesses distinct characteristics, and in industrial practice, they are often combined to achieve complementary strengths. Pyrometallurgical processes offer advantages in terms of high throughput and large-scale application, while hydrometallurgical processes demonstrate superior controllability and lower energy consumption [8,9]. These processes, along with their technical features and challenges, form one of the central themes of this review. In practice, the recovery of PGMs from SACs faces multiple challenges, including the deposition of contaminants and the oxidation of PGMs at high temperatures during catalyst deactivation, coupled with the encapsulation of PGMs within the cordierite. Notably, pyrometallurgical techniques utilizing base metal collectors (e.g., Fe, Cu) can effectively transfer PGMs into a metallic phase. This process reduces PGM oxides while achieving preliminary enrichment of PGMs [10]. Among current mainstream pyrometallurgical processes, the use of plasma smelting with an iron collector to recover PGMs from SACs is a significantly advantageous technology. Its core strengths lie in its large processing capacity, high PGM collection efficiency (≥ 99%), environmentally friendly collection process, and suitability for large-scale industrial production. A key product of this process is Fe−Si−PGMs alloy. This alloy represents a typical example of the challenges encountered in PGM recovery, as the presence of silicon in this alloy leads to the formation of a dense Fe-Si matrix, which exhibits strong chemical inertness. This inertness significantly increases the difficulty of subsequent hydrometallurgical leaching of PGMs [11,12]. Current primary treatment methods for Fe−Si−PGMs alloy include direct hydrometallurgical leaching, alkaline roasting, pyrometallurgical slagging, direct hydrometallurgical desilication, and in situ mechanochemical leaching [13]. These processes provide crucial technical references and foundational support for the efficient recovery of PGMs from Fe−Si−PGMs alloy.

Throughout the entire process of research, development, optimization, and monitoring of PGM recovery technologies, accurate and reliable analytical techniques are essential. However, the accurate quantification of PGMs remains inherently difficult due to their extremely high unit value, ultra-low concentrations in feed materials, and the high abundance and compositional complexity of matrix elements [14,15,16]. For instance, during the quantitative analysis of PGMs in Fe−Si−PGMs alloy or other PGM-bearing iron ore, the massive presence of matrix elements can severely interfere with the precise measurement of trace PGMs. Thus, analytical methodology constitutes another critical challenge in the PGM recycling chain. To overcome this challenge, researchers have developed various methods for PGM determination, many of which have made substantial contributions to PGM analysis. However, as with any analytical techniques, each approach is characterized by its own strengths and limitations. Currently, common methods for PGM determination include fire assay, high-resolution continuum source graphite furnace atomic absorption spectrometry (HR-CS GFAAS), X-ray fluorescence spectrometry (XRF), total reflection X-ray fluorescence spectrometry (TXRF), glow discharge mass spectrometry (GDMS), fusion methods, spectrophotometry, electrochemical techniques, and inductively coupled plasma optical emission spectrometry (ICP OES) [16,17,18,19]. In practical applications, the choice of method or a combination thereof is typically dictated by the specific requirements of the scenario.

In summary, confronted by the dual pressures of scarce primary PGM resources and the imperative for resource recovery from waste streams, recycling PGMs from SACs is critical for resource sustainability. This review focuses on two interconnected themes: the processes for PGM recovery from SACs and the associated challenges. It systematically collates fundamental PGM properties and resource availability, then critically examines pyrometallurgical and hydrometallurgical routes for SAC recycling, delineating their technical principles, research advances, and technological characteristics. Particular emphasis is placed on the fundamental properties of the highly inert Fe−Si−PGMs alloy, the main recovery processes, and their respective characteristics, as this alloy represents a significant challenge in the recovery flowsheet. Furthermore, the analytical techniques underpinning the entire recovery chain are reviewed, with discussions on the applicability and limitations of different methods, addressing another key challenge in achieving reliable PGM quantification. Through a thorough synthesis and comparative analysis of existing technologies, this work seeks to provide theoretical insights and technical guidance for developing more efficient, economical, and environmentally friendly PGM recovery and analytical methods, thereby advancing technological progress and fostering the sustainable development of the secondary PGM resource recycling industry.

## 2. Properties, Resources, and Applications of PGMs

### 2.1. Physical and Chemical Properties

**Figure 1:**
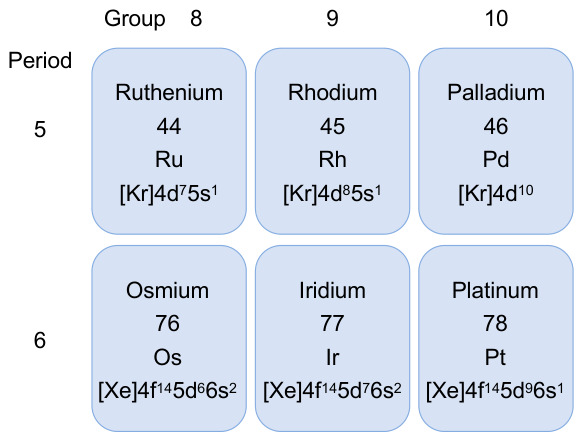
The positions of PGMs in the periodic table.

As illustrated in Figure 1, the six PGMs are all located in the d-block of the periodic table, specifically occupying groups 8, 9, and 10 in the fifth and sixth periods. A defining characteristic of these elements is the sequential filling of d orbitals with the same principal quantum number (*n*) as the atomic number increases, endowing PGMs with similar physical and chemical properties. The valence electron configurations for the fifth-period PGMs are 4d^7^5s^1^, 4d^8^5s^1^, and 4d^10^, while those for the sixth period are 5d^6^6s^2^, 5d^7^6s^2^, and 5d^9^6s^1^. Generally, the valence electron configuration of an atom significantly influences its chemical behavior. Configurations with completely filled or completely empty valence electron orbitals possess lower energy, thus conferring greater chemical stability. Among PGMs, the valence configurations of Pd (4d^10^), Os (5d^6^6s^2^), and Ir (5d^7^6s^2^) align with this fully filled state, which partially accounts for the stability inherent to PGMs [20,21].

However, from a broader perspective, the valence electron orbitals of PGMs exhibit irregular filling patterns. This phenomenon is likely attributable to energy level interleaving in atoms with high nuclear charge, where the energy levels of (*n* − 1)d orbitals and *n*s orbitals become comparable. This proximity creates a strong tendency for electrons from the *n*s orbital to transfer to the (*n* − 1)d orbital. This irregular electronic configuration also underpins the chemical stability of PGMs and their resistance to corrosion and oxidation in chemical reactions. PGMs demonstrate excellent stability in common inorganic acids, such as hydrochloric, sulfuric, and nitric acids. While Pd can dissolve in hot concentrated nitric acid, the other elements remain largely inert. At room temperature, PGMs are stable in air and oxygen, except for Os when in powder form. Furthermore, the partially filled d orbitals of PGMs enable the effective adsorption of reactants and the formation of coordination bonds. This process facilitates lowering of the reaction activation energy, promoting a more reactive state of the reactants and thereby imparting exceptional catalytic properties [2]. A prominent example is the hydrogen evolution reaction via water splitting catalyzed by Pt, where this characteristic is distinctly observable [22].

### 2.2. Resource Status

The global distribution of PGM resources is highly uneven, primarily dictated by geological and mineralization conditions, which have resulted in the concentration of high-grade deposits in limited countries and regions. This imbalance is reflected not only in geographical distribution but also in ore grade and mining economics. PGM resources are mainly located in southeastern Africa, northeastern Europe, northern Asia, and the north-central part of Northern America, where ore grades are generally high and mining is economically feasible [23].

According to the United States Geological Survey (USGS) 2024 report, global PGM reserves are estimated at approximately 71,000 tonnes. The United States, Canada, Russia, South Africa, and Zimbabwe rank as the top five countries in terms of PGM reserves, accounting for 1.15%, 0.44%, 7.75%, 88.73%, and 1.69% of the global total, respectively [24]. It is particularly noteworthy that South Africa, Russia, and Zimbabwe collectively hold over 98% of global PGM reserves, underscoring their extreme geographical concentration and posing significant implications for the security of the global PGM supply and market stability.

### 2.3. Application Sectors

**Figure 2:**
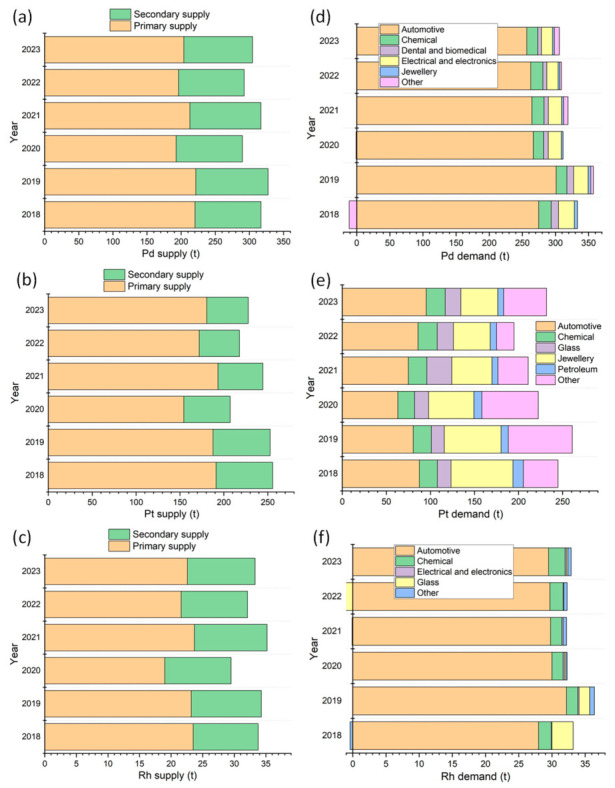
(**a**–**c**) The global supply status of Pd, Pt, and Rh, respectively; (**d**–**f**) the global demand status of Pd, Pt, and Rh, respectively [25].

As illustrated in Figure 2, based on data from the Johnson Matthey PGM market reports (2018–2023), the global supply and demand dynamics for PGMs exhibit distinct characteristics [25]. For Pd, the total primary mine supply is 207.9 tonnes, with the recycled supply contributing 100.1 tonnes. The recycled share of 32.5% highlights the significant role of recycling in the Pd supply chain. The demand structure is heavily dominated by automotive catalysts, accounting for 88% of the annual demand, followed by the electronics industry (6.5%), chemical industry (5.6%), biomedical applications (2.5%), and jewelry (1%). The overwhelming PGM demand from automotive catalysts, driven by increasingly stringent global vehicle emission standards, far exceeds that from all other applications. Regarding Pt, the total supply is 234.3 tonnes, with a recycled contribution of 54.5 tonnes (23.3%). This recycled share is 9.2 percentage points lower than that of Pd. Its demand exhibits a more diversified profile: while automotive catalysts remain the largest sector at 34.6% of total demand, the glass manufacturing (7.8%) and chemical (8.9%) industries show relatively higher demand shares, demonstrating the broad utility of Pt in industrial manufacturing. This structural difference primarily stems from the irreplaceable role of Pt in specific applications, such as high-temperature industrial catalysts and glass fiber production. The supply and demand pattern for Rh presents another distinct picture. With a total supply of 33.1 tonnes and a recycled share of 10.8 tonnes (32.6%), its recycled share is comparable to that of Pd. On the demand side, automotive catalysts hold absolute dominance at 90.3%, followed by the chemical industry (5.7%), glass manufacturing (1.9%), and electronics (0.5%). The concentration of Rh demand in automotive catalysts is notably higher than that for Pd and Pt, which is closely related to its unique catalytic reduction performance [26,27].

**Figure 3:**
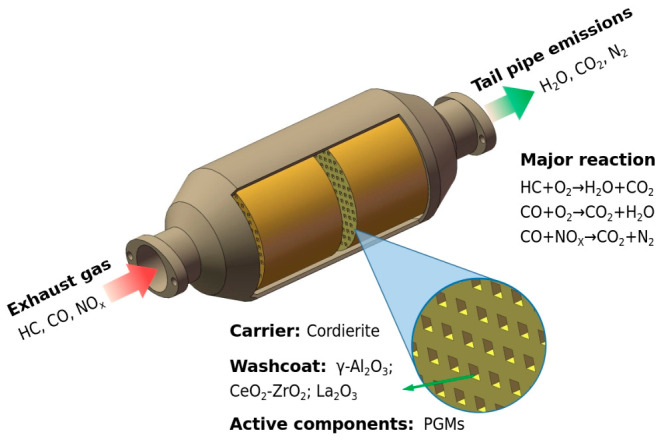
Structural schematic diagram of an automotive catalyst. Adapted from Ref. [26].

Automotive catalysts represent the largest application sector for Pd, Pt, and Rh. With continuous growth of the global vehicle fleet, the environmental pollution caused by vehicle emissions has become increasingly severe. Concurrently, heightened global emphasis on environmental protection has led to progressively stricter vehicle emission standards. To effectively reduce harmful gas emissions, automotive catalysts have emerged as a critical solution. PGMs play a pivotal role within these catalysts, primarily facilitating catalytic conversion reactions that transform harmful gases into harmless substances [13]. As illustrated in Figure 3, a typical automotive catalyst is primarily composed of a cordierite substrate, a washcoat, and active components. The cordierite provides structural support and acts as the framework for the catalyst, while the washcoat (main composition: γ-Al_2_O_3_; oxygen storage material: CeO_2_-ZrO_2_; additive: La_2_O_3_) enhances performance by improving thermal stability and dispersing the active components. Pd, Pt, and Rh are the active components, each targeting specific pollutants for directional catalysis. Pt and Pd catalyze the oxidation of CO and hydrocarbons, converting them into CO_2_ and H_2_O. Rh is primarily employed to catalyze the reduction of NO_x_, converting it into N_2_ and H_2_O [27,28].

Taking China as an example, the current vehicle parc has reached approximately 300 million units, forming a vast automotive consumer market. With the rapid development of the automotive industry and the accelerated pace of vehicle replacement, the number of end-of-life vehicles (ELVs) reached about 16.8 million in 2023. These ELVs represent a significant reservoir of PGMs. Assuming an average PGM content of 2 g per vehicle, the total PGM content amounts to roughly 33.6 tonnes. These data not only highlight the importance of SACs as a secondary resource but also underscores the substantial potential for recycling these metals to alleviate the supply–demand imbalance for PGMs [3,29,30].

Furthermore, the recycling of SACs holds multifaceted significance. From an environmental protection perspective, the recycling process effectively mitigates the pollution of soil, water sources, and the atmosphere by heavy metals and hazardous substances contained in discarded catalysts. Regarding resource utilization, the recovery of PGMs decreases dependence on primary mineral deposits and enhances resource efficiency. In terms of sustainable development, this initiative provides crucial support for achieving green and low-carbon development goals, contributing to the establishment of a resource-efficient and environmentally friendly society. In the future, with continuous advancements in recycling technologies and the gradual improvement of policy frameworks, the recycling of SACs is poised to play an increasingly vital role in resource security, environmental protection, and economic development [31,32].

## 3. Recovery Processes for SACs

### 3.1. Hydrometallurgical Processes

#### 3.1.1. Typical Leaching

In the field of PGM recovery from SACs, hydrometallurgical leaching serves as a critical pathway, achieving efficient metal extraction through liquid-phase chemical reactions. Conventional chemical leaching, as a core hydrometallurgical technology, operates through a selective dissolution mechanism. It utilizes the coordination/oxidation reaction characteristics of specific chemical reagents with PGMs to extract these metals from the catalyst substrate. Common lixiviants include cyanide-based solutions and combinations of hydrochloric acid with oxidizing agents.

Cyanide leaching is a chemical process based on the formation of stable cyanide complexes with PGMs. Its core mechanism relies on the strong complexing ability of cyanide to efficiently dissolve and separate PGMs from spent catalysts. Common cyanide lixiviants, such as sodium cyanide or potassium cyanide, react with PGMs under appropriate conditions to generate a series of soluble cyanide complexes, e.g., [Pt(CN)_4_]^2−^, [Pd(CN)_4_]^2−^, and [Rh(CN)_6_]^3−^. Cyanide leaching offers several advantages, the most notable being its high leaching efficiency and excellent selectivity for PGMs. However, the high toxicity of cyanides imposes stringent safety requirements for both the environment and operators during use, and the subsequent treatment costs are relatively high. Consequently, the application of cyanide leaching necessitates careful consideration of its potential risks and cost-effectiveness [33,34].

Leaching using hydrochloric acid combined with oxidants is another effective method for dissolving PGMs. In this process, hydrochloric acid provides the necessary acidic environment, while the oxidant promotes the oxidation of PGMs into soluble chloro-complexes, such as [PtCl_6_]^2−^, [PdCl_4_]^2−^, and [RhCl_6_]^3−^. Common oxidants include HNO_3_, NaClO_3_, Cl_2_, and H_2_O_2_. Compared to cyanide leaching, the HCl/oxidant method generally produces less toxic effluents, presents a lower potential environmental hazard, and offers sufficient efficiency to meet the requirements for the industrial recovery of PGMs. However, challenges remain. For instance, while the HCl/HNO_3_ system is highly efficient in PGM leaching, the use of HNO_3_ may lead to NO_x_ emissions, posing potential threats to the environment and operator health. Although the HCl/NaClO_3_ combination mitigates some environmental concerns compared to HNO_3_, risks of Cl_2_ emission persist, and the system exhibits strong corrosiveness to equipment, increasing maintenance costs. By contrast, H_2_O_2_ possesses strong oxidizing properties, and its reaction products are environmentally benign [35,36]. Such green oxidants warrant further in-depth research and application in the future, guided by the principles of sustainable metallurgy.

In summary, hydrochloric acid/oxidant leaching holds significant value in PGM recovery. However, different oxidant combinations present various trade-offs. While pursuing high recovery rates, it is essential to fully consider the environmental impact and sustainability of the process.

#### 3.1.2. Bioleaching

Bioleaching technology, as an environmentally friendly method for PGM recovery, has garnered significant attention in recent years [37]. Karim et al. [38] employed ultrasonication-assisted pretreatment combined with the cyanogenic bacteria *Pseudomonas fluorescens* and *Bacillus megaterium* to recover PGMs from SACs. The pretreatment using nitric acid under ultrasonication effectively removed metals such as copper and zinc that compete with PGMs for cyanide, thereby enhancing the efficiency of the subsequent bioleaching process. Experimental results demonstrated that in a two-step bioleaching process, *Pseudomonas fluorescens* achieved leaching efficiencies of 38% for Pt, 44% for Pd, and 91% for Rh under conditions of pH 9 and a pulp density of 1% (*w*/*v*). Under the same conditions, *Bacillus megaterium* achieved leaching efficiencies of 35%, 41%, and 82% for Pt, Pd, and Rh, respectively. Further research confirmed that the cyanogenic bacteria *Pseudomonas fluorescens* and *Bacillus megaterium* can effectively leach PGMs by producing cyanide, which forms soluble complexes with PGMs [39].

However, bioleaching technology faces practical challenges, including low leaching efficiency with prolonged recovery times, stringent microbial growth constraints (e.g., pH, temperature, nutrients), and the need for strict control over cyanide generation to prevent environmental contamination despite its biodegradability [40]. Future research should focus on developing more efficient and sustainable bioleaching processes.

#### 3.1.3. Photocatalytic Leaching

In recent years, photocatalytic technology has garnered increasing attention in the field of PGM resource recovery due to its unique electron transfer mechanisms. This technology utilizes electron-hole pairs generated upon photoexcitation of semiconductor materials to dissolve PGMs under mild reaction conditions, offering a viable strategy for the recycling of SACs [41].

Chen et al. [42] employed TiO_2_ as a photocatalyst to dissolve PGMs from SACs under ultraviolet irradiation, where the core of the process lies in selecting an appropriate photocatalyst and solvent. In their study, highly oxidizing radicals generated on the TiO_2_ surface exhibited a sufficient redox potential to overcome the chemical inertness of PGMs. Experimental results further showed that a mixed solvent system of acetonitrile and dichloromethane (volume ratio 3:1) synergistically enhanced the leaching efficiency of Pt, Pd, and Rh from SACs.

Photocatalytic leaching offers advantages of low energy consumption and mild reaction conditions, typically operating at room temperature and atmospheric pressure. While demonstrating potential for PGM recovery, this technology remains limited in reaction efficiency, stability, and scalability. Future efforts should prioritize overcoming these constraints and developing environmentally friendly reaction media [43].

#### 3.1.4. Solvent Extraction Separation

In hydrometallurgical processes, the efficient separation of PGMs from leachates represents a crucial yet challenging step, primarily due to their highly similar chemical properties, which complicates their distinction and separation in solution. Furthermore, leachates typically contain various impurity metals, which adds to the complexity of the separation process. Successful separation not only enables the independent recovery of different metals but also provides the necessary foundation for subsequent high-purity metal production, which is crucial for meeting the modern industrial demand for high-purity PGMs. Solvent extraction is an effective method for metal separation based on the differential distribution coefficients of metal ions between immiscible phases. This technique holds significant importance in the recovery of Pd, Pt, and Rh [5,26].

In practical applications, commonly used extractants mainly include organophosphorus acids, thiol derivatives, and ionic liquids. For instance, Yamada et al. [44] developed a series of dialkylamino-modified thiodiphenol extractants for the selective recovery of Pd^2+^ and Pt^4+^ from SAC leachates. Among these, 2,2′-bis(dihexylaminomethyl)-6,6′-thiobis(4-*t*-butylphenol) demonstrated excellent selectivity, achieving recoveries of 99.5% for Pd^2+^ and 99.3% for Pt^4+^ from the leachate, while recovery rates for impurity metals were below 3%. These extractants form stable complexes with PGMs, enabling effective separation. Additionally, ionic liquids have garnered attention as novel extractants due to their unique physicochemical properties, such as low volatility and high thermal stability. Firmansyah et al. [45] employed trioctyldodecyl phosphonium chloride (P_8,8,8,12_Cl) as an extractant to selectively recover Pd from SAC leachates. A Pd extraction efficiency of 99% was achieved within 10 min, followed by stripping using thiourea with a stripping efficiency of 90%.

Solvent extraction technology offers significant advantages in PGM recovery, such as high selectivity and recovery rates. However, the technique also presents challenges, including the selection and regeneration of extractants, the stability of the organic phase, and environmental concerns. Therefore, future research should focus on the development of novel extractants and the optimization of extraction processes to enhance the efficiency and sustainability of solvent extraction.

#### 3.1.5. Adsorption Separation

Adsorption separation is a method that utilizes the surface adsorption properties of adsorbents to separate Pd, Pt, and Rh ions. This technology has attracted significant attention due to its high efficiency and environmental friendliness. Commonly used adsorbents primarily include ion-exchange resins, activated carbon, metal–organic frameworks (MOFs), and biosorbents, with ion-exchange resins being the most widely applied [5].

Torrejos et al. [46] synthesized a series of thiacrown ether-based polydentate adsorbents. The study involved constructing a thiacrown ether diol skeleton containing 2 to 4 sulfur atoms, followed by the preparation of microporous resins using epoxy cross-linking technology. This material was employed for the selective recovery of Pt and Pd from SAC leachates. Experimental results indicated that these resins exhibited high selectivity for Pt and Pd adsorption, while showing markedly lower adsorption rates for alkali metals. Furthermore, Hong et al. [47] designed a polyethylenimine (PEI)-functionalized spinning fiber material based on cellulose nanofibers. This biosorbent possessed a porous structure and a high PEI content (32.5 wt%, rich in –NH_2_ groups), achieving a maximum Pt adsorption capacity of 417.6 mg/g after 24 h of adsorption in a pure Pt solution. When tested in a simulated SAC leachate, the adsorbent achieved simultaneous recoveries of 90% for Pt and 80% for Pd, while showing minimal adsorption for impurity metals such as Ni, Fe, and Mn.

Adsorption separation technology offers notable advantages in the recovery of Pd, Pt, and Rh, such as high selectivity and environmental compatibility. However, certain aspects of this technique require further attention, such as adsorbent stability, regeneration, capacity, and selectivity. Future research should focus on developing novel adsorbent materials and optimizing adsorption processes to improve both the efficiency and sustainability of the separation.

#### 3.1.6. Other Separation Techniques

Selective precipitation is another widely used hydrometallurgical separation technique. It involves the addition of specific precipitating agents to form insoluble precipitates with target metal ions, thereby achieving metal separation. Due to its simplicity and cost-effectiveness, selective precipitation has been extensively applied in the recovery of Pd, Pt, and Rh. Common precipitating agents include hydroxides, sulfides, and halides, which react with PGM ions to form insoluble precipitates. For example, Ilyas et al. [48] employed NH_4_Cl as a precipitating agent to recover Pt from a leachate, achieving a recovery rate exceeding 98%. This technique offers notable advantages, such as straightforward operation, low cost, and broad applicability. However, challenges remain, including the purification of precipitates, as well as the dosage and selection of precipitating agents.

Membrane separation is an emerging technique that utilizes specific membrane materials for the effective separation of Pd, Pt, and Rh. For example, the separation of Pt, Pd, and Rh can be achieved using polymer inclusion membranes containing P_8,8,8,12_Cl. The core of membrane separation technology lies in the selection of membrane materials and the optimization of membrane performance. By adjusting parameters such as composition, pore size, and surface charge, the separation efficiency can be enhanced. Furthermore, membrane separation can be combined with other methods, like solvent extraction and adsorption, to achieve more efficient metal recovery [49].

Metal displacement is a traditional separation method based on the reduction potential of PGMs. It involves using a more reactive metal (e.g., iron or zinc) as a reductant to displace Pd and Pt from solution. Rh can be selectively displaced by adding specific reducing agents. The critical factors for successful displacement include controlling the dosage of the reductant and the reaction conditions to achieve optimal results. Following displacement, subsequent steps such as washing, impurity removal, and calcination are typically required to further improve the purity of the recovered PGMs [50].

Solvent extraction, selective precipitation, adsorption, membrane separation, and metal displacement all play established roles in PGM recovery. In practical applications, a suitable separation strategy depends on factors such as target metal concentrations, leachate composition, and cost. The rational integration of multiple technologies can lead to the efficient separation and purification of PGMs.

### 3.2. Pyrometallurgical Processes

#### 3.2.1. Base Metal Collection

**Figure 4:**
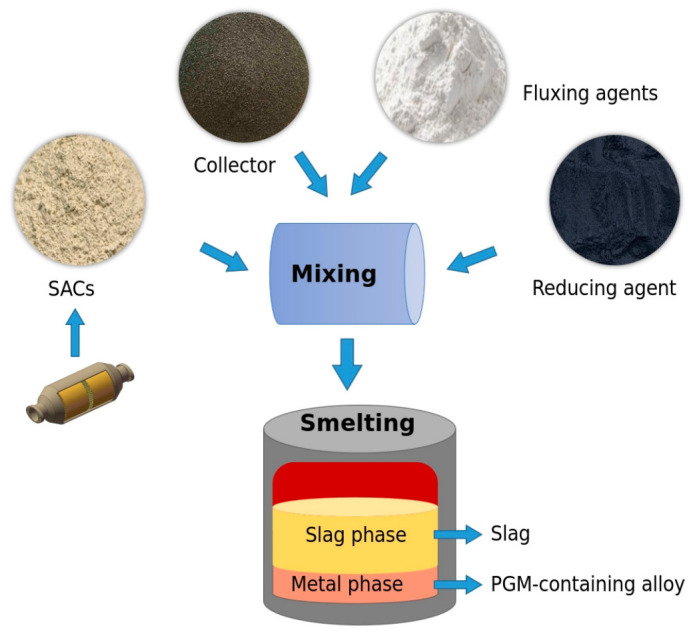
Schematic diagram of PGM recovery from SACs using a collector. Adapted from Ref. [51].

Metal collector-based recovery is one of the most common pyrometallurgical methods. This process involves mixing SACs with a collector metal and fluxing agents (e.g., lime, borax) and subjecting the mixture to high-temperature smelting. During this process, PGMs are concentrated into a metallic phase, achieving directional enrichment by leveraging the physical property differences between the metal and slag phases (as shown in Figure 4). Collector selection is typically based on factors such as PGM affinity, melting point, and environmental friendliness. Commonly used collectors include lead, copper, nickel, iron, bismuth, and sulfur, each with distinct characteristics, as detailed below [51,52].

Lead was one of the earliest metals used for PGM collection. In an arc furnace or blast furnace, lead oxide is reduced to lead, which alloys with PGMs, while the catalyst substrate reports to the slag phase. Lead collection offers operational simplicity, low-temperature operation, and established downstream refining. However, it suffers from drawbacks such as a lengthy processing cycle, lower recovery rates for Rh, and potential environmental and occupational health hazards associated with lead volatilization.

Copper collection has attracted significant attention due to its high recovery rates and lower environmental impact. Copper exhibits strong affinity for PGMs and effectively concentrates them during smelting. For example, Tanaka Kikinzoku Kogyo K.K. employs a copper collection technology where SACs are mixed with copper oxide, flux, and a reductant and smelted in a sealed furnace. The PGMs are concentrated into the copper phase and subsequently recovered via electrolysis, achieving a total PGM recovery rate of up to 98%. This technology can leverage existing non-ferrous metal smelting infrastructure, significantly reducing additional capital investment. Nonetheless, it also has disadvantages, such as a relatively long production cycle and high overall costs.

Nickel and nickel sulfide are often used simultaneously as collectors for PGM recovery from SACs. This process offers high recovery rates and selectivity, but the handling and recovery of sulfides are complex. Furthermore, sulfur-containing gases may be generated during smelting, posing potential environmental challenges and increasing the complexity and cost of off-gas treatment.

In recent years, bismuth collection has also attracted attention. Bismuth shows strong affinity for PGMs and can form alloys with them at relatively low temperatures, enabling effective PGM enrichment. This characteristic offers potential advantages in terms of energy consumption and cost. However, bismuth collection technology is currently still in the laboratory research stage and has not yet been commercially industrialized.

Iron and iron oxides serve as low-cost, environmentally benign, and highly effective collectors, efficiently incorporating PGMs into the iron phase under high-temperature conditions. For example, using iron powder as the collector, carbon as the reductant, and with additions of CaO, Na_2_O, CaF_2_, and Na_2_B_4_O_7_, >99% of PGMs were collected at 1300–1400 °C. This approach demonstrates the high collection efficiency achievable with iron-based systems, although some formulations rely on additives that raise environmental concerns. By contrast, iron collection has been successfully adapted in industrial settings using plasma smelting technology, which offers a more environmentally benign collection route with improved energy efficiency (see Section 3.2.4).

#### 3.2.2. Alkaline Roasting

Alkaline roasting primarily employs alkalis (e.g., KOH, NaOH) and alkali metal salts (e.g., Li_2_CO_3_, NaClO_3_, NaHSO_4_) as fluxes, which chemically react with the components of SACs at elevated temperatures to facilitate PGM recovery.

Specifically, the alkaline roasting treatment of PGMs from SACs typically follows two principal pathways. The first involves converting PGMs into corresponding alkali metal salts (e.g., Na_2_PtO_3_, Li_2_PtO_3_) through alkaline roasting, followed by leaching with common inorganic acids to extract the PGMs. For instance, Kuzuhara et al. [53] employed lithium salts (Li_2_CO_3_ or LiF) to roast SACs, which promoted the formation of PGM oxide salts Li_2_PdO_2_, Li_2_PtO_3_, and LiRhO_2_ for Pd, Pt, and Rh, respectively. The study found that Li_2_CO_3_ yielded better roasting results than LiF or a mixture of Li_2_CO_3_ and LiF. After roasting in air with Li_2_CO_3_ at 800 °C for 2 h, followed by leaching with concentrated HCl (12 mol/L), a leachate containing 94.9% Rh, 97.5% Pd, and 100% Pt was obtained. The second pathway involves treating the SAC substrate with alkalis or alkali metal salts to expose the PGMs, thereby enhancing subsequent leaching efficiency. For example, Trinh et al. [54] roasted SACs with NaOH at 600 °C with a sample-to-NaOH mass ratio of 1:1 for 1 h. This was followed by leaching the PGMs using a mixed solution of HCl and NaClO_3_ at 90 °C for 2 h, ultimately achieving leaching efficiencies of 97.5% for Pt, 98.8% for Pd, and 98.2% for Rh.

Alkaline roasting offers the advantages of simple processing and relatively mild conditions, while its industrial application requires addressing limitations such as severe equipment corrosion, high alkali consumption, and the complexity of subsequent leachate treatment.

#### 3.2.3. High-Temperature Chlorination

The high-temperature chlorination process, also known as chlorination-volatilization or chloridizing roasting, operates on the core principle of selectively reacting PGMs or their carrier materials with chlorine gas or chlorinating agents at elevated temperatures. This reaction forms volatile chlorides, thereby achieving effective separation of PGMs from the catalyst substrate.

In this process, SACs are typically mixed with chlorinating agents (e.g., NaCl, CaCl_2_, KCl) and roasted at a temperature within 650–900 °C. During roasting, volatile chlorides are evolved and subsequently condensed, achieving concentration of the PGMs. For instance, Xie et al. [55] successfully achieved efficient recovery of Pt, Pd, and Rh by mixing SACs with NaCl powder and introducing chlorine gas for chlorination at 650 °C. The chlorinated residue was then leached with 1 mol/L HCl aqueous solution at 90 °C for 1 h, achieving leaching efficiencies exceeding 97%, 99%, and 90% for Pt, Pd, and Rh, respectively.

The high-temperature chlorination process features a simple flowsheet, low energy consumption, and a high Rh recovery rate, without requiring complex pretreatment. However, it presents challenges such as equipment corrosion, toxic chlorine gas handling, and stringent environmental requirements, which necessitate careful mitigation in practice [56].

#### 3.2.4. Plasma Smelting Iron Collection

While various base metal collectors have been explored, iron-based collection has gained particular industrial prominence. Among iron-based approaches, plasma smelting iron collection has emerged as a mainstream technology, adopted by leading global SAC recycling companies such as Johnson Matthey (UK), Heraeus (Germany), and Sino-Platinum Metals Co., Ltd. (China) for industrial-scale production [56,57].

The plasma smelting iron collection process offers distinct advantages. Specifically, magnetite (Fe_3_O_4_) is used as the collector, metallurgical coke powder (C) as the reducing agent, and the smelting temperature is 1500–1600 °C. Only approximately 10% CaO is added to form an environmentally benign quaternary CaO–SiO_2_–Al_2_O_3_–MgO slag, which fully utilizes the cordierite substrate (2MgO•2Al_2_O_3_•5SiO_2_) and avoids adding harmful additives such as Na_2_B_2_O_4_ and CaF_2_. The pure cordierite has a high melting point of approximately 1900 °C and does not melt alone under the above temperature, but it undergoes structural decomposition in the presence of CaO to form molten slag, releasing the encapsulated PGMs [58,59]. Second, inexpensive and environmentally benign iron achieves collection efficiencies of 99.31%, 99.14% and 97.22% for Pd, Pt and Rh, respectively. Furthermore, plasma smelting delivers rapid, high-intensity heating that instantly reaches metal phase-transformation temperatures, cutting energy use, while its tunable redox environment optimizes PGM enrichment and curbs harmful emissions [11,60].

However, plasma smelting iron collection technology also faces several limitations. A crucial issue is that the Fe−Si−PGMs alloy obtained during the process exhibits strong chemical inertness, which represents a central challenge in the PGM recovery flowsheet [12]. Therefore, understanding the fundamental properties of this alloy (detailed in Section 4) and developing effective downstream processing strategies are of critical importance.

Table 1 compares the technical characteristics, advantages, and limitations of different PGM recovery processes for SACs. In industrial applications, suitable single or combined recovery processes are commonly selected according to technical feasibility, economic efficiency, and environmental performance.

## 4. Basic Properties of Fe−Si−PGMs Alloy

As introduced in Section 3.2.4, Fe−Si−PGMs alloy is the key intermediate product generated during plasma smelting iron collection. Its strong chemical inertness makes it a pivotal challenge in the overall PGM recovery chain. Detailed characterization of the chemical composition, phase constitution, and elemental distribution of the alloy is therefore essential for understanding the origin of this inertness and for designing effective downstream processing strategies. This section provides such an analysis.

### 4.1. Chemical Composition and Phase Constitution

As shown in Table 2, Fe−Si−PGMs alloy is primarily composed of two matrix elements, ten impurity elements, and three PGM elements. The matrix elements are Fe and Si. The impurity elements include P, Mn, Al, Ni, Ti, Ca, V, Cr, Mg, and Cu, while the PGM elements are Pd, Pt, and Rh. Fe is the predominant matrix element, with a content as high as 75.48%. This is attributed to the use of iron, which exhibits a high affinity for PGMs during the alloy formation process. The Si content is 15.07%, second only to Fe. This silicon originates primarily from the cordierite substrate of the SACs. Under the high-temperature reducing atmosphere, SiO_2_ is reduced to Si, which then alloys with Fe to form a highly corrosion-resistant matrix [61,62]. P has a content of 4.33%, mainly derived from engine lubricants and gasoline, and is one of the key factors leading to catalyst deactivation [63]. Furthermore, the presence of impurity elements such as Mn, Al, Ni, Ti, and Mg can also be traced to the cordierite. During plasma smelting, these elements are reduced but not fully incorporated into the slag, leaving trace impurities in the alloy.

**Figure 5:**
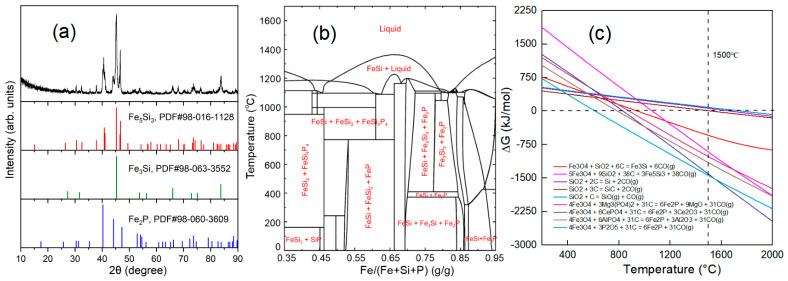
(**a**) XRD pattern of Fe−Si−PGMs alloy; (**b**) Phase diagram of Fe−Si−P ternary alloy (P = 4%) [62]; (**c**) ∆G–T curves of key carbothermal reduction reactions.

The alloy is dominated by Fe_5_Si_3_, Fe_3_Si, and Fe_2_P (Figure 5a). The Fe–Si–P ternary diagram (Figure 5b) predicts that within the Fe composition range of 70–83%, the stable phases are FeSi_3_, FeSi, and Fe_2_P, corresponding well with the XRD data. Thermodynamic calculations (Figure 5c) indicate that the initial temperatures for the reduction of SiO_2_ by C to form Si or SiO both exceed the furnace temperature (1500–1600 °C). SiC forms above 1530 °C, and its subsequent decomposition also requires temperatures beyond the furnace temperature [64]. By contrast, the presence of Fe_3_O_4_ significantly lowers the reaction temperature, enabling the reduction of SiO_2_ and the simultaneous formation of Fe_5_Si_3_ and Fe_3_Si at approximately 1060 °C, suggesting that the process is thermodynamically more favorable [65,66]. In SACs, phosphorus is mainly present as Mg_3_(PO_4_)_2_, CePO_4_, AlPO_4_, and P_2_O_5_, which react with Fe_3_O_4_ and C to form Fe_2_P at an initial temperature of approximately 880 °C, indicating that this reaction readily proceeds [67,68]. Although Fe_2_P exhibits poor corrosion resistance, the substantial formation of Fe-Si imparts strong chemical inertness to the alloy matrix.

### 4.2. Elemental Occurrence and Distribution

**Figure 6:**
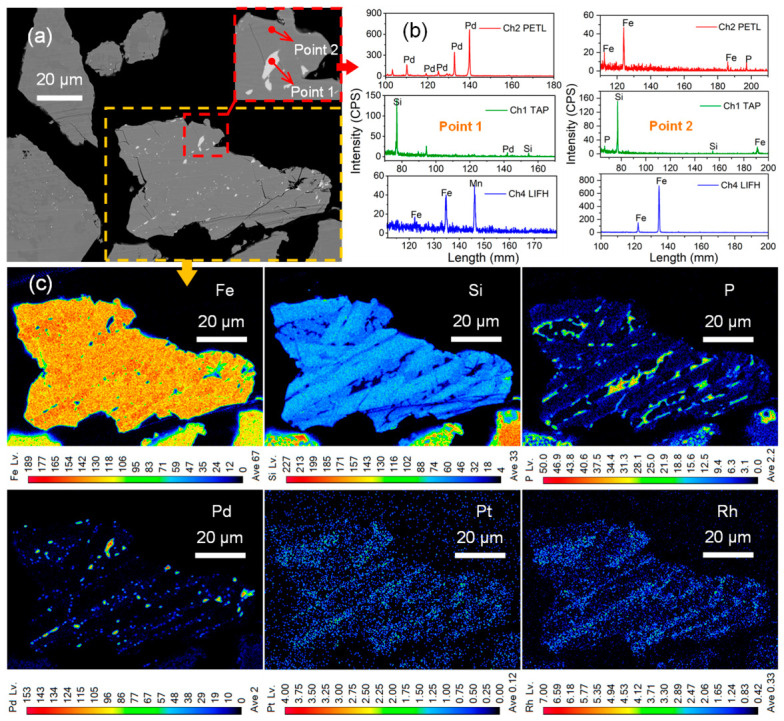
(**a**) Microstructure of Fe−Si−PGMs alloy; (**b**) Electron probe spectra of Points 1 and 2; (**c**) Distribution of main elements [62].

Microstructural analysis of Fe−Si−PGMs alloy (Figure 6a,b) reveals irregular particle shapes. The bright regions (Point 1) are rich in PGMs, chiefly Pd, whereas the dark regions (Point 2) consist mainly of Fe, Si, and P. Elemental mapping (Figure 6c) further shows that Fe and Si are the most extensively and uniformly distributed matrix elements. This is attributed to their high abundance and tendency to form intermetallics, such as Fe_3_Si and Fe_5_Si_3_. The impurity P does not simultaneously combine with both Fe and Si but preferentially associates with Fe to form Fe_2_P, which segregates at the grain boundaries. Among PGMs, Pd primarily segregates at the grain boundaries, whereas Pt and Rh are distributed uniformly within the matrix. Overall, the distribution of Pd, Pt, and Rh across the alloy is relatively homogeneous [13,62]. The inherent high chemical stability of PGMs, combined with their encapsulation within a highly inert Fe-Si matrix, significantly exacerbates the difficulty of leaching PGMs from Fe−Si−PGMs alloy [42,69]. This combination thus represents a central challenge in the PGM recovery flowsheet, motivating the development of specialized treatment technologies discussed in Section 5.

## 5. PGM Recovery Processes from Fe−Si−PGMs Alloy

### 5.1. Direct Hydrometallurgical Leaching

Direct hydrometallurgical leaching involves treating Fe−Si−PGMs alloy directly with lixiviants without specifically targeting silicon removal. Tong et al. [70] employed a two-stage leaching process to treat Fe−Si−PGMs alloy (96.16% Fe, 0.25% Si, and 0.3% PGMs). The first stage (room temperature, 1.7–1.8 mol/L H_2_SO_4_, S/L = 1:10, 30 min) used a substoichiometric acid dosage to partially dissolve Fe and produce a low-acidity FeSO_4_ solution suitable for Fe recovery via freeze crystallization, meanwhile enriching PGMs by approximately 16 times. The second stage (75 °C, 2 mol/L H_2_SO_4_, S/L = 1:50, 4 h) applied excess acid to deeply remove Fe from the residue, generating a high-grade PGM concentrate. After the two-stage process, PGMs were concentrated by an average factor of 70.57, reaching a final grade of 21.18% with less than 0.2% dissolution loss. This process demonstrates excellent performance in PGM concentration. However, it involves a lengthy flowsheet and produces substantial leachate, while also being restricted to Fe−Si−PGMs alloy with low silicon content [71].

**Figure 7:**
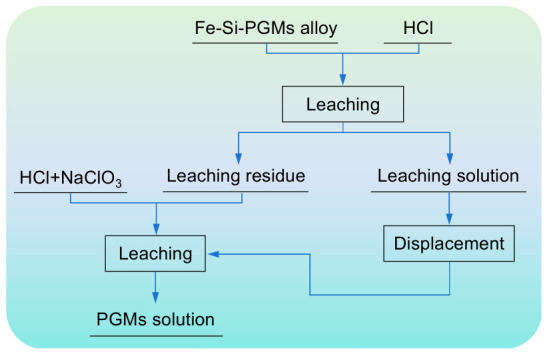
Two-stage leaching of Fe−Si−PGMs alloy.

Wu et al. [72] investigated direct hydrometallurgical treatment of high-silicon Fe−Si−PGMs alloy (70% Fe, 10–15% Si, 4–6% PGMs). As shown in Figure 7, the overall process comprises two main stages: bulk Fe removal via HCl leaching, followed by oxidative leaching of PGMs. The first stage uses 6 mol/L HCl at 75 °C for 4 h, removing about 50% of Fe. Under these conditions, a small fraction of the PGMs also dissolved, with Pd being the most soluble, followed by Pt, while Rh remained largely undissolved. These dissolved PGMs were recovered by cementation. In the second stage, the residue was subjected to oxidative leaching in acidic NaClO_3_ solution under optimized conditions: 6 mol/L HCl, 250 rpm, L/S = 10:1, 80 °C, 2 h, and NaClO_3_-to-alloy of 1:1. This yielded leaching efficiencies of 62.1% (Pd), 57.3% (Pt), and 25.4% (Rh). These results demonstrate the extremely high corrosion resistance of Fe−Si−PGMs alloy, providing a valuable foundation for subsequent research. However, the leaching efficiencies for PGMs still have considerable room for improvement.

### 5.2. Alkaline Roasting for Desilication

Alkaline roasting for desilication involves the reaction of alkaline substances with silicon in Fe−Si−PGMs alloy to form soluble silicates, thereby facilitating silicon removal.

**Figure 8:**
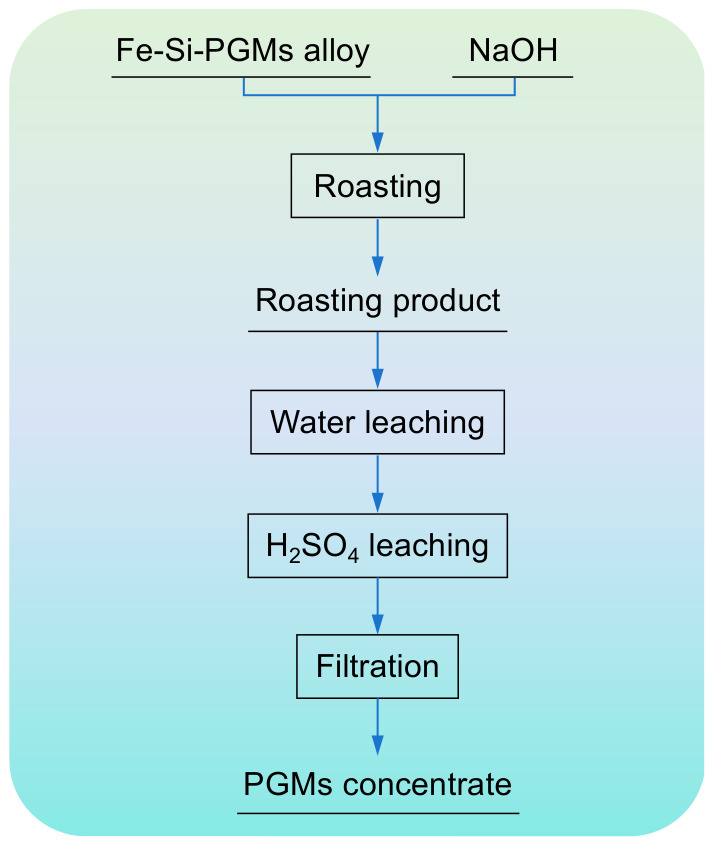
Alkaline roasting for silicon removal.

Dong et al. [73] developed a roasting-leaching method to concentrate PGMs from Fe−Si−PGMs alloy, with optimal conditions at 600 °C, 120% NaOH-to-alloy, and 2 h. Roasting converted the alloy mainly into Na_4_SiO_4_, NaFeO_2_, and Fe_2_O_3_. Subsequent water and dilute H_2_SO_4_ leaching removed NaFeO_2_ and Fe_2_O_3_ (Equations (1)–(5)), respectively, leaving a residue enriched with PGMs (Figure 8). This residue contained 23.4% PGMs, achieving a 9-fold enrichment from the original alloy.

(1) $$\mathrm{Si}+4\mathrm{NaOH}={\mathrm{Na}}_{4}{\mathrm{SiO}}_{4}+2H_{2}$$

(2) $$2\mathrm{Fe}+6\mathrm{NaOH}=2\mathrm{Na}{\mathrm{FeO}}_{2}+3H_{2}+2{\mathrm{Na}}_{2}O$$

(3) $$2\mathrm{Na}{\mathrm{FeO}}_{2}={\mathrm{Fe}}_{2}O_{3}+{\mathrm{Na}}_{2}O$$

(4) $$2\mathrm{Na}{\mathrm{FeO}}_{2}+(n+1)H_{2}O={\mathrm{Fe}}_{2}O_{3}\cdot {\mathrm{nH}}_{2}O+2\mathrm{NaOH}$$

(5) $${\mathrm{Fe}}_{2}O_{3}\cdot H_{2}O+3H_{2}{\mathrm{SO}}_{4}={\mathrm{Fe}}_{2}{\left({\mathrm{SO}}_{4}\right)}_{3}+{4H}_{2}O$$

However, this process still has room for further optimization. First, the removal efficiencies for Fe and Si are relatively low. Enhancing their removal requires intensifying the alkali metal roasting process, which inevitably risks oxidizing a portion of the PGMs, thereby increasing the potential for PGM loss. Secondly, the process does not achieve the direct dissolution of PGMs, adding extra process steps compared to direct leaching methods.

### 5.3. Pyrometallurgical Slagging

**Figure 9:**
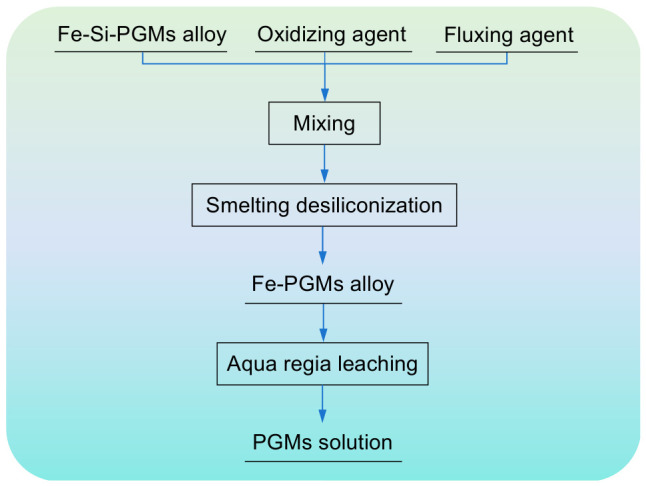
Pyrometallurgical slagging for desiliconization combined with aqua regia leaching.

The core principle of the pyrometallurgical slagging desilication combined with aqua regia leaching process lies in oxidizing the silicon in Fe−Si−PGMs alloy to form SiO_2_. The SiO_2_ then combines with a slagging agent to enter the slag phase, achieving effective silicon removal. The resulting Fe-PGMs alloy is subsequently leached with aqua regia to obtain a PGM-containing solution (Figure 9).

Li et al. [74] employed a plasma furnace as the smelting unit, using Fe_2_O_3_ as the oxidant and CaO as the slagging agent to remove silicon from Fe−Si−PGMs alloy. The study found that increasing the oxidant dosage, smelting temperature, and duration effectively promoted the desilication process. The desilication rate initially increased with CaO up to 20% of the raw material mass, but declined with further addition due to increased melt viscosity. The optimal conditions were determined as 140% of theoretical Fe_2_O_3_, 20% CaO, 1600 °C, and 120 min, which reduced alloy silicon from 10% to 0.5% to facilitate subsequent PGM leaching.

In 2023, Yang et al. [75] developed a green slag refining process using a CaO–Al_2_O_3_–Fe_2_O_3_ slag system to remove >99% of major impurities (Si, Al, Ca, Mg, Ti) from Fe−Si−PGMs alloy, followed by complete alloy dissolution via aqua regia leaching. Pyrometallurgical slagging desilication operates through selective oxidation and alloy composition adjustment. Fluxes (CaO, SiO_2_, Al_2_O_3_) form low-melting-point slags with FeO_x_. To ensure a green process, fluorides and chlorides should be avoided. The impurities Si, Al, Ti, Ca and Mg, whose oxygen affinities exceed that of Fe, are oxidized to oxides (Equations (6)–(10)) and report to slag, while FeO_x_ is reduced to metallic Fe and enters the alloy.

(6) $$\mathrm{Si}+\frac{2}{x}{\mathrm{FeO}}_{x}=\frac{2}{x}\mathrm{Fe}+{\mathrm{SiO}}_{2}$$

(7) $$2\mathrm{Al}+\frac{3}{x}{\mathrm{FeO}}_{x}=\frac{3}{x}\mathrm{Fe}+{{\mathrm{Al}}_{2}O}_{3}$$

(8) $$\mathrm{Ti}+\frac{2}{x}{\mathrm{FeO}}_{x}=\frac{2}{x}\mathrm{Fe}+{\mathrm{TiO}}_{2}$$

(9) $$\mathrm{xCa}+{\mathrm{FeO}}_{x}=\mathrm{Fe}+\mathrm{xCaO}\;$$

(10) $$\mathrm{xMg}+{\mathrm{FeO}}_{x}=\mathrm{Fe}+\mathrm{xMgO}\;$$

The pyrometallurgical slagging process produces no liquid or gaseous waste, offering environmental benefits, and when combined with aqua regia leaching, it enhances the recovery efficiency of PGMs from Fe–Si–PGMs alloy. However, it is limited by higher energy consumption, potential PGM loss in slag, and the environmental drawbacks of aqua regia leaching.

### 5.4. Hydrometallurgical Desilication Leaching

Hydrometallurgical desilication combined with aqua regia leaching first disrupts the matrix structure to liberate PGMs, followed by aqua regia leaching of the resulting residue.

In 2024, Kuzas et al. [76,77] investigated the leaching behavior of Fe–Si–PGMs alloy (76.8% Fe, 11.9% Si, 1.4% PGMs) in various acidic solutions. Using HCl or H_2_SO_4_ followed by aqua regia achieved <40% PGM recovery, indicating ineffective breakdown of the inert Fe-Si matrix. Tests with HCl–HF mixtures showed that higher HCl promoted Fe leaching but suppressed Si volatilization, while increased HF enhanced both Fe dissolution and Si release. Elevated temperature significantly improved Fe extraction but had a minor effect on Si leaching.

**Figure 10:**
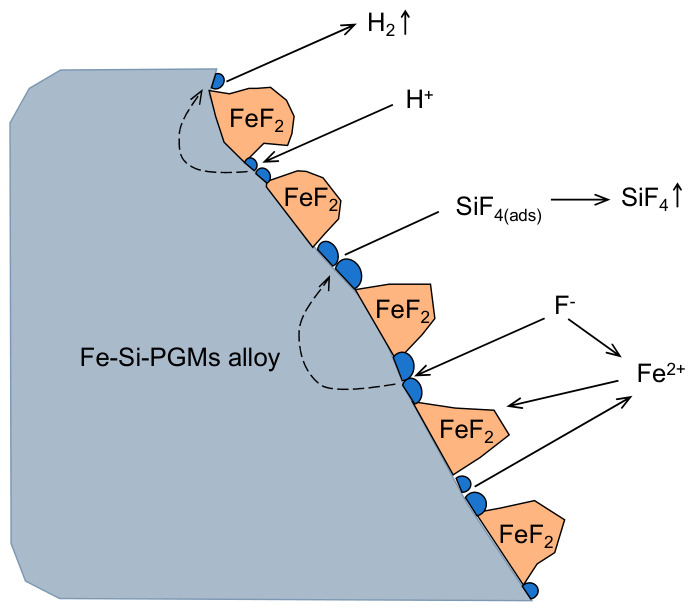
Schematic diagram of the leaching mechanism of Fe–Si–PGMs alloy in HCl and HF mixed solution [76].

**Figure 11:**
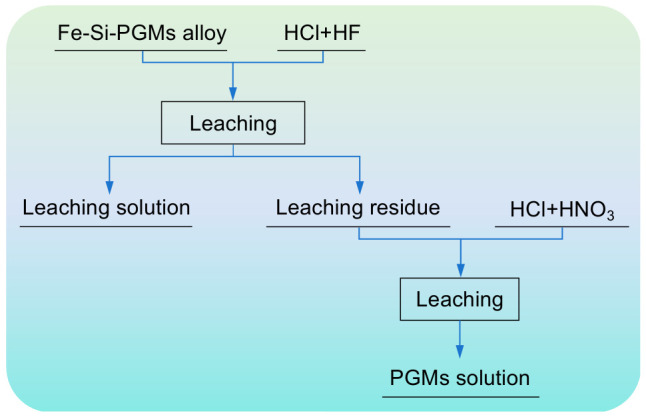
Hydrometallurgical desilication combined with aqua regia leaching.

The mechanism of leaching the Fe–Si–PGMs alloy matrix with a mixed HCl–HF solution is illustrated in Figure 10. During leaching, the alloy matrix reacts with HCl and HF, generating Fe^2+^ and SiF_4_ gas (Equation (11)), which explains the promotional effect of HCl on Fe leaching and the simultaneous promotion of both Fe and Si leaching by HF. However, free HF can react with Fe^2+^ to form FeF_2_ precipitate (Equation (12)). This precipitate forms a dense film on the surface of the unreacted alloy, hindering contact between the lixiviant and the alloy and the diffusion of reaction products, leading to a decrease in Si leaching efficiency. Leaching kinetics studies revealed that Fe leaching transitioned from chemical reaction control to diffusion control, while Si leaching remained diffusion-controlled throughout, primarily due to the diffusion resistance caused by the FeF_2_ film. Through the shrinking core model and full factorial experimental analysis, the optimal leaching conditions were identified: 4.36 mol/L HCl, 6.93 mol/L HF, 90 °C, and 80 min. Under these conditions, leaching efficiencies for both Fe and Si could reach 95%. Subsequent treatment of the residue with aqua regia enabled complete dissolution of the PGMs, with the specific process flow shown in Figure 11 [76,77].

(11) $${\mathrm{Fe}}_{x}{\mathrm{Si}}_{y}+2\mathrm{xHCl}+4\mathrm{yHF}=x{\mathrm{FeCl}}_{2}+\mathrm{ySi}F_{4}↑+(x+2y)H_{2}↑$$

(12) $${\mathrm{FeCl}}_{2}+2\mathrm{HF}=\mathrm{Fe}F_{2}↓+2\mathrm{HCl}\;$$

Hydrometallurgical desilication combined with aqua regia leaching provides an effective process for destructing the inert alloy matrix to liberate PGMs, achieving their recovery from Fe–Si–PGMs alloy. Nevertheless, concerns remain regarding the use of HF, an extremely corrosive acid that poses serious threats to human health. Additionally, the SiF_4_ generated during leaching adversely affects human health and the environment [78,79].

### 5.5. In Situ Mechanochemical Leaching

Mechanochemistry is an interdisciplinary technique that utilizes mechanical energy input to initiate or enhance chemical reactions. It has demonstrated significant potential in fields like materials synthesis, mineral processing, and solid waste valorization. Its characteristics, including mechanical force-induced lattice distortion, increased specific surface area, and accelerated mass transfer, provide a novel approach for the activation and efficient leaching of highly inert materials [80].

**Figure 12:**
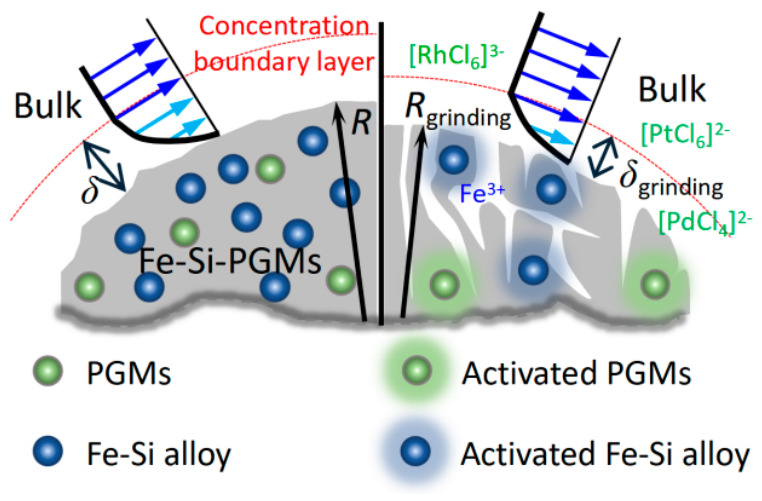
Schematic illustration of the in situ mechanochemical extraction mechanism of PGMs from Fe–Si–PGMs alloy [62].

Liu et al. [62] designed an HCl-based ternary lixiviant system for the in situ mechanochemical leaching of Fe–Si–PGMs alloy. Under optimized conditions (L/S of 30:1, 2 mol/L HCl, 0.75 mol/L FeCl_3_, 2.5 mol/L H_2_O_2_, 800 rpm, 4 h), a comprehensive PGM leaching efficiency of 98% was achieved. This system employs FeCl_3_ and H_2_O_2_ as green oxidants, avoiding the generation of harmful NO_x_ associated with traditional aqua regia leaching. The mechanical force serves multiple functions: it simultaneously achieves particle size reduction and surface activation of the alloy, disrupting the Fe-Si matrix and exposing fresh reactive interfaces. It also accelerates lixiviant mass transfer and reduces activation energy, thereby overcoming thermodynamic and kinetic barriers to PGM dissolution (Figure 12) [13]. While in situ mechanochemical leaching offers significant advantages, challenges remain for its scale-up. The positive pressure generated by gas evolution during the reaction and the demanding equipment performance requirements under high rotational speeds necessitate further in-depth research.

A comparison of the above processes for recovering PGMs from Fe–Si–PGMs alloy is summarized in Table 3.

## 6. Analytical Methods for PGMs

The accurate quantification of PGMs represents another critical challenge in the recovery chain, given their trace levels, high value, and complex matrices. Analytical errors can lead to misestimation of resources, loss of process control, and significant economic or environmental consequences. Therefore, the selection or combination of analytical methods must be based on the specific application scenario, taking into account the principles, advantages, and limitations of each technique (Table 4) [61,81].

### 6.1. Fire Assay

Fire assay, a classical metallurgical analytical method, applies pyrometallurgical principles to analytical chemistry. It involves separating PGMs from impurities through high-temperature fusion, followed by quantitative determination via weighing or other analytical techniques. This method offers advantages such as strong adaptability to diverse samples and accurate, reliable results. However, the fire assay process is relatively complex, requiring specialized equipment and skilled operators over extended durations. It also poses environmental and health risks due to volatile lead and slag emissions [81,82]. Therefore, when employing fire assay, appropriate measures must be implemented to minimize environmental impact and ensure operator safety.

### 6.2. HR-CS GFAAS

HR-CS GFAAS is a method that determines PGM concentration by using a high-resolution continuum source to excite the PGM atoms in the sample and measuring the absorption signal at specific wavelengths. Its advantages include high sensitivity and low detection limits, and it is capable of measuring elements at ppm levels. However, this technique has limitations for multi-element simultaneous determination due to constraints on spectral windows and the requirement for consistent thermal behavior of elements. Sample matrices can also interfere with analytical results, and the equipment cost is relatively high [16,83].

### 6.3. XRF

XRF is a non-destructive analytical technique based on the interaction of X-rays with the sample. Its fundamental principle is that when primary X-rays irradiate a PGM sample, inner-shell electrons of atoms are excited, leading to energy-level transitions and the emission of characteristic secondary X-rays. The energy and intensity of these fluorescent X-rays correlate with the type and concentration of elements in the sample. By detecting these signals, qualitative and quantitative analyses can be performed. XRF offers the advantages of simple operation, rapid analysis, and low cost. However, it has limitations in detection limits and accuracy, especially in complex matrices where matrix effects can cause deviations in PGM content determination [84]. TXRF is an improved method developed from conventional XRF. It uses a smooth reflector to focus X-rays onto the sample, providing more concentrated irradiation, thereby enhancing signal intensity and signal-to-noise ratio. This method significantly lowers detection limits and improves analytical accuracy. However, TXRF still faces challenges with matrix interference and accuracy, and the equipment cost is relatively high [85,86]. XRF-based analytical methods offer significant advantages for the rapid detection of PGM types and contents in solid waste materials such as SACs.

### 6.4. GD-MS

GD-MS is an analytical technique that combines a glow discharge source with a mass spectrometer. Its fundamental principle involves using ions generated from ionized inert gas under high voltage to bombard the sample surface, causing sputtering and subsequent ionization of atoms from the sample. These ions are then collected and detected by the mass analyzer. GD-MS offers advantages such as high sensitivity, low matrix effects, and a wide linear dynamic range. However, it also presents some limitations: PGM materials with poor conductivity require special treatment, increasing sample preparation complexity and contamination risk; the equipment cost is high, demanding specialized maintenance and operators; and it is primarily suitable for analyzing inorganic solids, with limited capability for liquid samples [87,88,89]. Therefore, in practical application, GD-MS is mainly used for analyzing the purity of high-purity solid PGM materials.

### 6.5. Fusion

The basic principle of fusion involves converting PGMs in the sample into water-soluble salt compounds. During fusion, the PGM-containing sample is mixed with strongly oxidizing alkaline reagents (e.g., NaOH, KOH, Na_2_O_2_) and heated to a high temperature. This process drives a chemical reaction that efficiently breaks down the structure of samples, converting solid PGMs into a soluble form [90]. The fusion method offers high efficiency, broad applicability, and excellent recovery rates for dissolving solid-phase PGMs. Its operation is relatively simple and cost-effective. However, it introduces impurities via strong alkalis and oxidants, requires skilled high-temperature operation, and causes crucible corrosion that potentially contaminates the sample [91,92].

### 6.6. Spectrophotometry

Spectrophotometry quantifies PGMs by measuring the absorbance of their characteristic colored complexes at specific wavelengths. For instance, Pt is determined by reacting Pt^4+^ with SnCl_2_ to form a yellow complex, measuring the absorbance at 403 nm, and calculating the concentration from a standard curve [93]. Zhao et al. [94] determined Rh by forming a violet complex between a rhodium-tin chloride anion and crystal violet cation, measuring the absorbance at 540 nm. Wang et al. [95] leached geological samples for Pd and Pt using a HCl–KClO_3_–NaCl–NH_4_HF_2_ mixture, preconcentrated the metals on thiol cotton, ashed the metal-loaded thiol cotton, dissolved the resulting residues, and finally determined the Pd and Pt contents spectrophotometrically. This method is relatively simple, requires no large instruments, and has low operational costs, offering advantages for field analysis. However, it suffers from interferences from other ions or organics in complex matrices, often requiring masking agents or pretreatment. Results are also sensitive to experimental conditions, demanding strict control for accuracy [96].

### 6.7. Electrochemical Voltammetry

Electrochemical voltammetry is a powerful technique for PGM analysis based on measuring the current at a working electrode during a potential scan in a solution containing electroactive species. Different electrode materials and scan modes enable the detection and quantification of target PGM ions [97]. For example, adsorption cathodic stripping voltammetry was used to determine Pt in oyster samples from coastal environments. Oyster ash was dissolved in an electrolyte containing H_2_SO_4_, formaldehyde, and hydrazine. A three-electrode system with a hanging mercury drop working electrode was used. After preconcentration at −0.3 V, a differential pulse scan from −0.5 V to −1.1 V reduced Pt, generating a current signal proportional to its concentration [98,99]. This method offers high sensitivity, low detection limits, and rapid operation, making it suitable for trace PGM analysis. Limitations include electrode surface fouling or poisoning by organics/impurities and significant matrix interference from factors like acidity and salinity, often requiring masking agents for mitigation [100].

### 6.8. ICP OES

**Figure 13:**
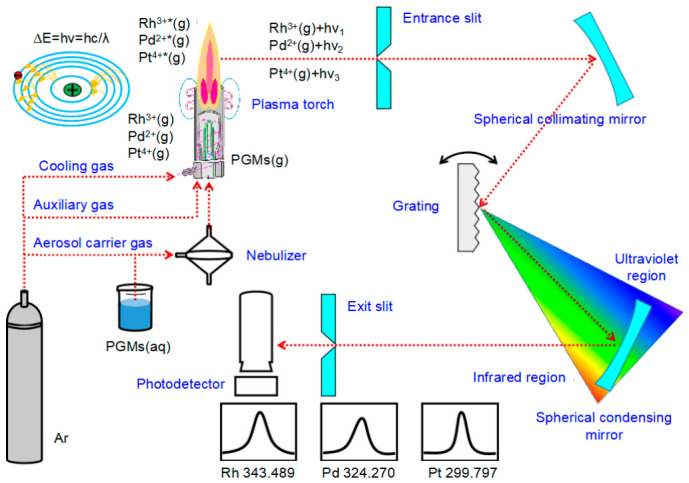
The process of analyzing PGMs using ICP OES (* Excited state).

ICP OES currently represents the most widely employed spectroscopic technique for PGM analysis. It operates by introducing a sample into a high-temperature plasma (8000–10,000 K), where atoms/ions are excited and subsequently emit characteristic light upon returning to lower energy states [101]. The intensity of this emitted light, measured at specific wavelengths, correlates with elemental concentration [102]. As shown in Figure 13, samples are nebulized into an aerosol, transported to the plasma, excited, and their emitted light is dispersed and detected. ICP OES offers advantages including multi-element capability, low detection limits, wide linear range, and minimal chemical interference, making it a primary method in PGM metallurgy. However, spectral overlap and matrix effects from complex sample compositions can compromise accuracy, necessitating effective interference mitigation strategies [103,104].

Common methods to mitigate these interferences include sample pretreatment, dilution, matrix matching, standard addition, and correction factor method. However, these approaches require careful attention to avoid introducing new interferents, potential PGM losses, or analyte contamination. Pretreatment can effectively remove matrix components, but attention must be paid to the risk of increased analytical error and PGM loss. Dilution is operationally simple but may reduce analytical sensitivity. While methods such as standard addition and matrix matching can improve measurement accuracy, they are limited by relatively complex and time-consuming [103,105]. The correction factor method offers a convenient and efficient mathematical approach; however, careful consideration must be given to the applicability and accuracy of the correction model [106,107]. For instance, Liu et al. [61] investigated matrix interferences in the analysis of PGMs in Fe–Si–PGMs alloy. They found that the complex matrix caused both spectral and non-spectral interferences. By establishing a mathematical correction model to compensate for matrix effects, the relative errors in the determination of Pd, Pt, and Rh were reduced from above 10.2% to within 5.5%.

## 7. Summary and Outlook

This review systematically examines PGM recovery from SACs, focusing on two interconnected challenges: Fe–Si–PGMs alloy processing and accurate PGM quantification.

In SAC recycling, pyrometallurgical and hydrometallurgical methods are typically combined: the former is commonly used to enrich PGMs and modify their chemical environment, while the latter is primarily employed for leaching and subsequent deep separation. In Fe–Si–PGMs alloy, the homogeneous distribution of Pd, Pt, and Rh within the inert Fe-Si matrix, combined with their inherent stability, results in strong chemical resistance. Current treatment approaches can be broadly categorized into desilication-leaching and non-desilication leaching. Desilication methods, including alkaline roasting, pyrometallurgical slagging, and hydrometallurgical desilication, significantly enhance PGM leaching rates. However, both alkaline roasting and slagging introduce additional energy-intensive pyrometallurgical steps and increase the risk of PGM loss. Hydrometallurgical desilication poses hazards to human health and the environment. By contrast, non-desilication leaching offers better environmental compatibility and lower energy consumption. However, direct hydrometallurgical leaching suffers from low PGM extraction efficiency due to matrix constraints. In situ mechanochemical leaching, which couples mechanical activation with chemical dissolution, enables effective disruption of the inert matrix and enhanced PGM extraction, although further research is required to optimize the process and reaction environment.

Accurate PGM quantification is complicated by diverse matrices and high precision demands. Each analytical method carries distinct strengths and limitations. Method selection requires comprehensive consideration of the sample matrix, costs, sample state, and analytical requirement. Fire assay effectively separates the matrix, offers strong sample adaptability, and provides high accuracy, but it involves complex procedures and poses environmental and health risks. HR-CS GFAAS demonstrates high accuracy, yet its instrumentation involves considerable cost. XRF-based techniques allow for rapid qualitative analysis, but their quantitative precision is relatively low. GD-MS is primarily applied to the detection of high-purity PGM products, although the equipment is relatively expensive. Fusion can effectively dissolve highly inert PGM materials, but the influence of substantial impurities must be considered. Spectrophotometry features straightforward operation; however, its measurements are prone to interference from coexisting substances. Electrochemical methods offer accuracy and speed, yet their applicability is limited by specific testing system requirements. ICP OES analysis features high detection efficiency and reliable accuracy, making it one of the most prevalent methods in PGM recovery. However, spectral interferences and matrix effects must be taken into consideration.

Future efforts should center on green extraction technologies that integrate technical, economic, and environmental criteria to establish an efficient, clean, and sustainable PGM recycling system.

Whole-process optimization and coupling: Strengthen the integration between pyrometallurgical enrichment and hydrometallurgical extraction–separation, and develop short-process, low-waste integrated flowsheets from SACs to high-value PGM products. Rather than optimizing individual unit operations in isolation, holistic flowsheet design must be emphasized, as industrial practice requires the seamless integration of dissolution, separation, and reduction steps [2]. Improve overall metal recovery, resource efficiency, and process economics by combing multiple metallurgical techniques (e.g., pyrometallurgy, hydrometallurgy, and electrochemistry). Moreover, process optimization should prioritize direct product preparation to eliminate intermediate solidification. Conversion of the PGM chloro-complex route from a ‘liquid–solid–liquid’ to a ‘liquid–liquid’ paradigm would enable direct production of catalyst precursors from leachate, thereby eliminating redundant reduction/redissolution steps, reducing process complexity, and minimizing wastewater generation. Concurrently, industrial-scale selective separation materials (e.g., extractants, ion-exchange resins, adsorbents) for PGMs, particularly for rhodium, warrant development, with particular attention to both stripping and elution efficiencies [8].

Efficient and green processing of Fe–Si–PGMs alloy: Prioritize novel techniques for disrupting the Fe-Si matrix and selectively extracting PGMs in an environmentally benign strategy. Key areas include: (a) advancing mechanochemistry, ultrasound, and microwave-enhanced methods, as well as microbial recovery, toward practical application; (b) developing green and efficient oxidation or ligand systems for PGM dissolution under mild conditions; and (c) optimizing pyrometallurgical desilication to lower energy and material use while minimizing PGM loss to slag. In developing green leaching agents, priority should be given to H_2_O_2_, free radicals, and high-valent cerium compounds as oxidants to eliminate the generation of toxic gases such as NO_x_ and Cl_2_. Concurrently, hydrochloric acid consumption can be reduced through the use of chloride salts (e.g., FeCl_x_, CuCl_2_) as alternative chlorine sources. Furthermore, the potential of vacuum metallurgy and mechanochemistry warrants further investigation, with a focus on equipment optimization, energy consumption reduction, and scale-up for industrial application [7,108].

Innovations in analytical techniques for PGMs in complex matrices: For samples such as PGM-containing materials and environmental samples, novel analytical methods should aim for simpler pretreatment, higher interference resistance, lower detection limits, and potential on-site/rapid detection. Concurrently, artificial intelligence and big data can be leveraged to optimize analytical strategies and calibration models, enhancing overall efficiency, accuracy, and reliability.

## Figures and Tables

**Table 1:** Comparison of mainstream PGM recovery processes from SACs.

Process	Principle	Advantages	Limitations	Performance	Ref.
**Hydrometallurgical**					
Cyanide leaching	Formation of stable cyanide complexes	High efficiency, excellent selectivity	High toxicity, costly waste treatment	96% Pt, 98% Pd, 91% Rh leaching	[33,34]
HCl + oxidant leaching	Oxidation to soluble chloro-complexes	Lower environmental hazard, industrial applicability	NO_x_/Cl_2_ risk, strong equipment corrosion	88–96% Pt, 85–99% Pd, 77–95% Rh leaching	[35,36]
Bioleaching	Cyanogenic bacteria produce cyanide	Environmentally friendly, mild conditions	Slow kinetics, low efficiency, strict microbial growth control	35–38% Pt, 41–44% Pd, 82–91% Rh leaching	[38,39]
Photocatalytic leaching	Photoexcited semiconductors generate electron–hole pairs	Low energy consumption, mild conditions	Low efficiency, limited scalability, solvent toxicity	98% Pt, 92% Pd, 85% Rh leaching	[41,42,43]
Solvent extraction	Differential distribution in immiscible phases	High selectivity, high recovery, industrial application	Extractant selection/regeneration, organic phase stability	99% Pd, 99% Pt recovery	[5,26,44,45]
Adsorption separation	Functionalized adsorbent (resins, MOFs, biosorbents)	High efficiency, environmentally friendly	Adsorbent stability/regeneration, low capacity	90% Pt, 80% Pd recovery	[5,46,47]
Selective precipitation	Formation of insoluble PGM precipitate	Simple, low cost, broad applicability	Precipitate purification, reagent dosage control	>98% Pt recovery	[48]
Membrane separation	Size exclusion/charge interaction of membranes	High selectivity, low energy consumption	High cost, easy fouling, strict feed requirements	96% Pd, 96% Pt, 99% Rh recovery	[49]
Metal displacement	Reduction via more reactive base metals	Simple process, low cost	Poor selectivity, secondary pollution	60% Pt, 100% Pd recovery	[50]
**Pyrometallurgical**					
Base metal collection	Alloying PGMs with collector	High throughput, industrial application, high efficiency	Long cycle, high energy consumption	93% Pd, 95% Pt, 97% Rh recovery	[51,52]
Alkaline roasting	Conversion of PGMs to soluble salts/exposure of encapsulated PGMs	Simple process, relatively mild conditions	Severe corrosion, high alkali consumption, complex leachate treatment	>98% Pt, >98% Pd, >95% Rh leaching	[53,54]
High-temperature chlorination	Formation of volatile PGM chloride	Simple flowsheet, high Rh recovery	Equipment corrosion, toxic chlorine, stringent safety	97% Pt, 99% Pd, 90% Rh leaching	[55,56]
Plasma smelting iron collection	Plasma smelting with Fe_3_O_4_ collector for PGM concentration	High efficiency, environmentally benign slag, no harmful additives	Fe−Si−PGMs alloy inertness	99% Pt, 99% Pd, 97% Rh recovery	[11,56,57,58,59,60]

**Table 2:** Main chemical composition of Fe−Si−PGMs alloy (wt%) [61].

**Element**	**Fe**	**Si**	**P**	**PGMs**	**Mn**	**Al**	**Ni**
Content	75.48	15.07	4.33	2.18	0.82	0.46	0.41
**Element**	**Ti**	**Ca**	**V**	**Cr**	**Mg**	**Cu**	**Others**
Content	0.30	0.24	0.17	0.17	0.09	0.09	0.19

**Table 3:** Comparison of PGM recovery processes from Fe–Si–PGMs alloy.

Process	Principle	Advantages	Limitations	Performance	Ref.
Direct hydrometallurgical leaching	Two-stage H_2_SO_4_ leaching to dissolve Fe selectively	High PGM enrichment, low dissolution loss	Long flowsheet, large leachate volume, only suitable for low-Si alloy	PGMs enriched 70-fold, final grade 21%	[70,71]
Direct hydrometallurgical leaching	HCl leaching to remove Fe, then NaClO_3_ oxidative leaching	Simple, low energy consumption	Long flowsheet, low PGM leaching	Pt 57%, Pd 62%, Rh 25% leaching	[72]
Alkaline roasting	Roasting with NaOH, then leaching to remove Fe and Si	Simple, moderate conditions, achieves PGM enrichment	Low Fe/Si removal efficiency, risk of PGM loss	PGMs enriched 9-fold, grade 23%	[73]
Pyrometallurgical slagging	Slagging of Si with Fe_2_O_3_/CaO, then aqua regia leaching	Green impurity removal, efficient impurity removal	High energy consumption, potential PGM loss, Cl_2_/NO_x_	Si reduced to 0.5%, complete PGM leaching	[74,75]
Hydrometallurgical desilication leaching	HCl–HF breaks Fe-Si matrix, then aqua regia leaching	Effective destruction of inert alloy matrix, high Fe/Si removal	Serious health and environmental risks, HF/Cl_2_/NO_x_	95% Fe, 95% Si, 100% PGM leaching	[76,77]
In situ mechanochemical leaching	Mechanical activation in HCl–FeCl_3_–H_2_O_2_ system	Short flowsheet, high PGM leaching, no HF/Cl_2_/NO_x_	Challenging scale-up, demanding equipment requirements	>99% PGM leaching	[13,62,80]

**Table 4:** Comparison of analytical methods for PGM quantification.

Method	Principle	Advantage	Limitation	Ref.
Fire assay	High-temperature fusion separation	Robust, accurate	Complex, toxic emissions	[81,82]
HR-CS GFAAS	Atomic absorption in graphite furnace	High sensitivity, low DL	Limited multi-element, matrix effects	[16,83]
XRF/TXRF	Characteristic X-ray fluorescence	Non-destructive, rapid	Moderate DL, matrix effects	[84,85,86]
GDMS	Glow discharge mass spectrometry	High sensitivity, low matrix effect	Requires solids, expensive	[87,88,89]
Fusion	Alkaline oxidative dissolution	High efficiency, good recovery	Impurity introduction, corrosion	[90,91,92]
Spectrophotometry	Absorbance of colored complexes	Simple, low cost, field-ready	Interferences, condition-sensitive	[93,94,95,96]
Electrochemical voltammetry	Current response during potential scan	High sensitivity, fast	Electrode fouling, matrix interference	[97,98,99,100]
ICP OES	Atomic emission in plasma	Multi-element, wide range, low DL	Spectral/matrix interferences	[61,101,102,103,104,105]

DL = detection limit.

## Data Availability

No new data were created or analyzed in this study. Data sharing is not applicable to this article.
